# Do Left-Handed Older Adults Have Superior Visual Memories?

**DOI:** 10.1177/00315125231185166

**Published:** 2023-06-22

**Authors:** Annukka K. Lindell

**Affiliations:** 1 Department of Psychology, Counselling and Therapy, 2080 La Trobe University, Melbourne, VIC, Australia

**Keywords:** memory, visual, verbal, left handedness, right handedness

## Abstract

On demanding visual memory tasks like the Rey Complex Figure Test and Recognition Trial (RCFT), left-handers often outperform right-handers and participants with mixed handedness. Left-handers’ apparent visual memory superiority develops during late childhood and early adolescence and is established by young adulthood. Though many studies have examined RCFT performance in older adults and found that visual memory deteriorates with age, investigations of the relationship between handedness and visual memory abilities in older adults have been scarce. In the present study I sought to determine whether a left-handed RCFT performance advantage would be evident among older adults. I examined RCFT and handedness data from 800 older adults (Females = 152, Males = 648; *M* age = 69.86, *SD =* 5.18 years; range 60–85 years), who took part in prior research (Whitehall II Phase 11 sub-study). Among these participants, handedness predicted both immediate and delayed RCFT recall, with left-handers outperforming both mixed- and right-handers and with performance unrelated to gender. The absence of a left-handed advantage for copy accuracy suggests that the effects observed for recall do not stem from differences in participants’ perceptual abilities and/or motor control. Instead, these data suggest that left-handers’ superior performances stem from their advantage for visual memory. As visual memory predicts both motor learning capacity and motor skill retention in older adults, these results have potentially important implications for rehabilitation efficacy.

## Introduction

Left handedness has long been linked to visuospatial processing advantages (Lokhurst, 1982). Indeed, a classic neurofunctional dichotomy holds that the left cerebral hemisphere is typically characterized by primary involvement in verbal/language processing whereas the right hemisphere is primarily involved in visuospatial processing (Corballis, 2003). Given predominant contralateral cortical control of the body’s motor and sensory processing, differences between the cerebral hemispheres’ cognitive processing proclivities have led to expectations that damage to the right cerebral hemisphere causes profound deficits in visuospatial functioning (e.g., De Renzi et al., 1977; Bartolomeo, 2021). Contralateral motor control, with primary right hemisphere involvement in left hand movement, implies that left handers may be right hemisphere dominant, with advantages in visuospatial processing (Fritsche & Lindell, 2019).

The Rey Complex Figure Test and Recognition Trial (RCFT; Meyers & Meyers, 1995), based on the classic Rey-Osterreith Complex Figure Test (Rey, 1941; Osterrieth, 1944), is a popular, robust measure of visuospatial abilities. The RCFT task assesses visuospatial organization, planning, and memory, by presenting participants with a complex geometric figure, asking them to copy it, and, later, without prior warning, to recall and draw the figure from memory. The incidental recall components of the RCFT are thought to minimize any verbal encoding of the Rey figure details (Casey et al., 1990).

Annett (1992) first demonstrated a relationship between handedness and RCFT performance, with strongly left-handed males outperforming right-handed and less strongly dominant left-handed males. For females, however, the best performance was observed among right handers with strong sinistral tendencies (i.e., weaker right handers), while left handers and strong right handers performed more poorly. This gender difference has been attributed to differences in strategies, with right-handed females presumed to have adopted more verbal strategies for recalling spatial information (e.g., Inglis & Lawson, 1982).

More recent research has similarly indicated a left-handed advantage for RCFT performance among both males and females (e.g., Rakhmavov & Dane, 2020). Rakhmavov and Dane (2020) studied Nigerian university students and used the Edinburgh Handedness Inventory (Oldfield, 1971) to establish handedness; they found that both male and female left-handers showed superior RCFT memory, with higher scores on both immediate and delayed recall than right-handers. Other researchers found that a family history of left-handedness was associated with better RCFT performance (D’Andrea & Spiers, 2005). Whilst D’Andrea and Spiers (2005) found no differences in Rey figure copy accuracy between right-handed females with and without left-handed relatives, immediate and delayed recall were superior for females who had at least one family member with left-handedness or ambidexterity. Similar findings have been consistently reported by other researchers (e.g., Casey et al., 1990; Weinstein et al., 1990), supporting the notion that brain organization favoring superior visuospatial abilities is associated with being left-handed and/or having left-handed relatives.

In keeping with broad trends in psychological research (Henreich et al., 2010), most studies examining the relationship between handedness and RCFT performance have assessed university students (e.g., Annett, 1992; D’Andrea & Spiers, 2005; Rakhmavov & Dane, 2020). In an exception to this trend, Karapetsas and Vlachos (1997) offered important developmental insight by examining children (age range 5.5–12.5 years) and determining handedness from five items on the Edinburgh Handedness Inventory (Oldfield, 1971). Contrary to findings with adults of a left-handed visuospatial advantage, younger children (ages 5.5 – 10.5 years) who showed a right hand preference produced superior RCFT performance even while there were no significant relationships between handedness and RCFT performance among older children (ages 10.5 – 12.5 years). Earlier research by the same group (Karapetsas & Vlachos, 1992) showed a pattern of increasing RCFT performance in left-handed children, adolescents and young adults (ages 5.5 – 20.5 years). Karapetsas and Vlachos’s (1992, 1997) findings imply that the visuospatial advantages that were often evident in research with left-handed adults (and right handers with a history of familial sinistrality) are not evident in youth and develop over time.

Of note in this discussion, visuospatial functioning tends to decline with age, deteriorating at a faster rate than other cognitive skills (e.g., Murre et al., 2013). Indeed, Murre et al.’s findings indicated that, while memory performance decreases 1–3% per year after age 25, visuospatial memory starts deteriorating earlier (age 18 onward) and declines at twice the rate of verbal memory decline. More recent findings indicate that visuospatial function (as assessed by the RCFT) is related to older adults’ responsiveness to motor rehabilitation therapy in that better visuospatial function indexes better motor skill retention in healthy older adults (*M* age = 70.38 years; Lingo VanGilder et al., 2021). Other research has similarly suggested that stronger visuospatial function is linked to better motor learning capacity in older adults (e.g., Lingo VanGilder et al., 2018). As such, visuospatial evaluation with the RCFT, a neuropsychological measure that is known to be less affected by language and cultural differences than other common measures (Zhang et al., 2021), may help predict long-term motor skill retention, allowing early identification of patients at risk for poor response to rehabilitation efforts.

Whilst research has examined RCFT performance in adults and older adults, I found no investigations of the relationship between handedness on RCFT performance in older adults. Given the potential for visuospatial functioning among older adults to predict their responsiveness to rehabilitation, there is both theoretical and clinical utility to establishing the relationship between handedness and visuospatial function in older adults. In the present study, I sought to determine whether the left-handed advantage evident in young adults (e.g., Annett, 1992; D’Andrea & Spiers, 2005; Rakhmavov & Dane, 2020) persists into older adulthood. As visual memory increases with increasing intelligence (i.e., intelligence quotient (IQ) test scores) (Gallagher & Burke, 2007) but declines with increasing age (Murre et al., 2013), I included age and intelligence as covariates in my modelling analyses in this investigation to control for their potentially confounding influence on memory performance. I drew RCFT and handedness data from participants in the Whitehall II Phase 11 sub-study, allowing examination of visuospatial memory performance in 800 older adults (aged 60 – 85). I anticipated that left-handers would demonstrate superior immediate and delayed recall performance on the RCFT in comparison with right-handers.

## Method

### Participants

For this study, I drew data from 800 participants (females = 152, males = 648; (*M* age = 69.86, *SD* = 5.18 years; range 60–85 years) from the Whitehall II study dataset (Phase 11; 2012–2016). The Whitehall II study is a prospective longitudinal investigation initially established in 1985 to investigate the factors underlying a social gradient in morbidity and mortality (the lower the employment grade the higher the morbidity and mortality rates). All civil servants employed in 20 London-based British Civil Service Departments, and aged 35–55 in 1985–1988 (*N* = 10,308 in Phase 1), were invited to take part, with a 73% response rate (74% for men and 71% for women). The study received ethical approval from the University of Oxford Central University/Medical Science Division Interdisciplinary Research Ethics Committee and the Health Research Authority NRES Committee South Central – Oxford B (details provided in Filippini et al., 2014).

This sub-study was conducted in Oxford and included a range of clinical and cognitive test measures with test administration lasting up to two hours and including assessments of handedness and visual memory (see Filippini et al., 2014, for the Whitehall II Phase 11 Imaging Substudy protocol). Researchers can apply for access to these data from the data custodians (Dementia Platforms UK: https://www.dementiasplatform.uk/) and can find information about the whole study and data sharing from University College London (https://www.ucl.ac.uk/epidemiology-health-care/research/epidemiology-and-public-health/research/whitehall-ii) and the Imaging Substudy from the UKRI grant Web site (https://gtr.ukri.org/projects?ref = G1001354#/tabOverview).

### Assessment Materials

#### Handedness

Participants’ handedness was assessed using the self-administered Briggs and Nebes (1975) handedness questionnaire. This measure examines participants’ preferred hand(s) for completing 12 tasks (e.g., “Indicate the hand you use to throw a ball to hit a target.”), with the questions drawn from Annett’s (1967) handedness inventory. The scoring of items in the Briggs and Nebes questionnaire permits assessing the strength of the participant’s hand preference by relying on a finer assessment of the degree of handedness than is obtained from a blunt binary “left/right” response. Each item is scored on a five point scale: “always” = 2 points, “usually” = 1 point, and “no preference” = 0 points; “left” answers are assigned negative values, and “right” answers are assigned positive values (item range −2 - +2). Summing the points for all 12 items results in a handedness score ranging from −24 (extreme left-handedness) to +24 (extreme right-handedness).

#### Rey Complex Figure Test and Recognition Trial (RCFT)

The RCFT asks participants to copy a complex geometric diagram (the “Rey Figure”) and then reproduce it from memory (Meyers & Meyers, 1995). The task requires many different cognitive abilities, including visuo-constructional skills and both short and long-term visual memory (Fillipini et al., 2014; Zhang et al., 2021).

##### Copy

For the copy task, participants were first provided with paper and a drawing implement, and a printout of the Rey Figure, and they were asked to reproduce the figure to the best of their ability, with no time constraints. Next, the participant’s copy was removed from view.

##### Immediate Recall

After a three-minute delay, the participant was provided with a fresh piece of paper and was asked to reproduce the figure to the best of their ability from memory.

##### Delayed Recall

Finally, after a delay of 30 minutes, the participant was provided with a fresh piece of paper and was asked to reproduce the figure again to the best of their ability from memory.

The participants’ three drawings of the Rey Figure (Copy, Immediate Recall, Delayed Recall) were scored according to the Meyers and Meyers (1995) manual, assessing the accuracy of the reproduction and placement of 18 specific design elements in the Rey Figure. Previous research has confirmed high levels of inter-rater reliability (*r*s > .88) for Rey Figure scoring using this method (e.g., Liberman et al., 1994).

##### Demographics

Demographic data, including the participants’ age, gender, and premorbid intelligence were assessed in a survey and with the Test of Premorbid Functioning (TOPF; Wechsler, 2011), for which standard scores were recorded.

### Statistical Analyses

The data were analyzed using generalized linear modelling to examine the influence of participants’ handedness and gender as predictors of RCFT performance, with age and TOPF IQ score entered as covariates. Separate regression models were conducted to assess copying, immediate recall, and delayed recall scores. Handedness was entered as a continuous variable (−24 - +24), and gender was entered as a categorical variable (female, male), in all models. The statistical significance level was set at p < .05, with all analyses performed using the Statistical Package for the Social Sciences (SPSS, version 28; IBM Corp, 2021. Armonk, NY).

## Results

### Participant Descriptives

**Figure 1.**
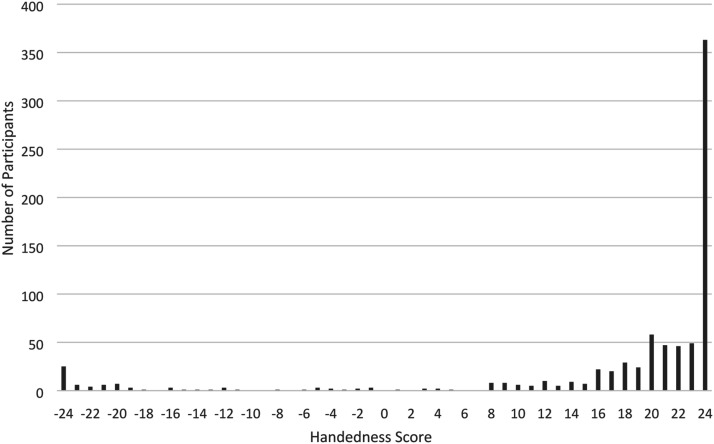
Demographics: Distribution of Handedness scores. *Note:* - 24 indicates extreme left handedness; +24 indicates extreme right handedness.

Three participants from the 800-participant dataset were excluded for incomplete data, resulting in a final sample of 797 for data analysis (152 females - 19.07%; 645 males - 80.93%). Participants’ mean age was 69.85 years (*SD* = 5.17; range 60–85), and their mean TOPF estimated premorbid intelligence score (IQ) was 118.45 (*SD* = 10.08; range 75–151). Consistent with the general population, these participants were predominantly strongly right-handed, with 45.5% of participants scoring the highest possible right-handed score (+24) and 3.1% of participants gaining the highest possible left-handed score (−24). According to Briggs and Nebes (1975), scores ranging from −24 - -9 are classed as left-handed, −8 - +8 are mixed handed, and +9 - +24 are considered right-handed. In the current sample there were (a) 62 left handers (7.77%), (b) 27 mixed handers (3.39%) and (c) 708 right handers (88.83%). Figure 1 illustrates the distribution of handedness scores across these participants.

There were no differences in premorbid intellectual ability between the three handedness groups [*F*(2, 794) = 2.23, *p* = .110], with similarly high mean TOPF IQ scores for left-handers (*M* = 120.46; *SD* = 8.39), mixed-handers (*M* = 115.77; *SD* = 8.84), and right-handers (*M* = 118.38; *SD* = 10.24). However there were differences in their ages [*F*(2, 794) = 4.69, *p* = .009], with post-hoc analysis indicating that, on average, left-handers (*M* age = 68.05; *SD* 4.66 years) were younger than right-handers (*M* age = 69.97; *SD* = 5.17 years) [*F*(1, 768) = 7.96, *p* = .005], and mixed-handers (*M* age = 71.03; *SD* = 5.41) [*F*(1, 87) = 6.96, *p* = .010]; right-handers and mixed-handers did not differ [*F*(1, 733) = 1.08, *p* = .298].

### Rey Complex Figure Test and Recognition Trial (RCFT) Analyses

Handedness did not predict RCFT copying performance (please refer to Table 1 for the results of the modelling analysis and Table 2 for descriptive data). Though the test comparing the full model to a model with intercept only was significant, χ^2^(63) = 167.15, *p* < .001, neither participant handedness nor gender predicted RCFT copying accuracy. The interaction between handedness and gender was similarly not significant. Both covariates were however significantly related to copying performance, with higher intelligence associated with greater copy accuracy, whilst increases in age were associated with decreases in copy accuracy.

**Table 1.** Results of the Generalized Linear Modelling Analysis for RCFT Copying.

		β	SE	95% CIs		*χ2*	*p*
				LL	UL		
Predictors	Handedness	−1.44	1.64	−4.64	1.77	33.42	0.759
	Gender	0.05	0.47	−0.87	0.96	0.65	0.419
	Handedness * gender	1.69	1.83	−1.9	5.27	21.01	0.397
Covariates	TOPF IQ	0.12	0.01	0.09	0.14	71.68	**<.001**
	Age	−0.12	0.03	−0.17	−0.64	19.99	**<.001**

*Note:* TOPF IQ = Test of Premorbid Functioning standard score.

**Table 2.** RCFT Means (and *SD*s) for Immediate Recall, Delayed Recall, and Copying, as a Function of Handedness (Left, Mixed, Right).

		Copying		Immediate Recall		Delayed Recall	
Handedness	*N*	Mean	SD	Mean	SD	Mean	SD
Left (−24 - -9)	62	31.98	3.32	18.52	5.92	17.41	6.59
Mixed (−8 - +8)	27	31.85	3.33	16.83	6.69	16.70	6.49
Right (+9 - +24)	708	31.49	4.01	15.43	6.56	15.08	6.22

In contrast handedness significantly predicted both immediate and delayed RCFT recall performance, even when controlling for age and TOPF IQ scores (please refer to Tables 3 and 4). For immediate recall, a test of the full model versus a model with intercept only was highly significant, χ^2^(63) = 175.79, *p* < .001. Results confirmed that handedness was a significant predictor of immediate recall, with left-handers gaining higher recall scores than mixed-handers who, in turn, gained higher recall scores than right-handers (please see Table 2). In contrast neither participant gender nor the interaction between handedness and gender, predicted immediate recall performance. Both covariates were highly associated with performance, with recall increasing with increases in TOPF IQ scores, but decreasing with increases in age.

**Table 3.** Results of the Generalized Linear Modelling Analysis for RCFT Immediate Recall.

		β	SE	95% CIs		*χ2*	*p*
				LL	UL		
Predictors	Handedness	10.81	4.05	2.89	18.75	58.82	**0.028**
	Gender	1.55	0.78	0.02	3.08	0.95	0.329
	Handedness * gender	2.72	3.03	−3.22	8.66	22.00	0.340
Covariates	TOPF IQ	0.14	0.23	0.10	0.19	39.60	**<.001**
	Age	−0.20	0.04	−0.28	−0.12	22.38	**<.001**

*Note:* TOPF IQ = Test of Premorbid Functioning standard score.

**Table 4.** Results of the Generalized Linear Modelling Analysis for RCFT Delayed Recall.

		β	SE	95% CIs		*χ2*	*p*
				LL	UL		
Predictors	Handedness	11.05	3.91	3.38	18.71	57.42	**0.037**
	Gender	1.32	0.76	−0.16	2.80	1.29	0.255
	Handedness * gender	4.89	2.93	−0.85	10.64	21.45	0.371
Covariates	TOPF IQ	0.13	0.02	0.09	0.17	35.42	**<.001**
	Age	−0.19	0.04	−0.27	−0.10	20.20	**<.001**

*Note:* TOPF IQ = Test of Premorbid Functioning standard score.

As indicated in Table 4, a similar pattern emerged for delayed RCFT recall, with a test of the full model versus a model with intercept only highly significant, χ^2^(63) = 162.44, *p* < .001. Handedness was a significant predictor of delayed recall performance, with left-handers again evidencing higher recall than either mixed- or right-handers (please refer to Table 2). In line with the immediate recall performance, neither participant gender nor the interaction between handedness and gender, influenced delayed recall performance. Consistent with the immediate recall results both covariates were again associated with performance, with delayed recall accuracy increasing with increases in IQ scores, and decreasing with increasing age.

## Discussion

In the present study, I sought to determine whether the left-handed advantage for visuospatial processing that has been observed in younger adults (e.g., Rakhmavov & Dane, 2020) would be evident for older adults. Analysis of data from 797 older adult participants in the Whitehall II Imaging sub-study confirmed that left-handers produced superior immediate and delayed RCFT recall performance in comparison with right- and mixed-handers, while participants’ gender was not associated with a performance difference. As handedness did not predict copy accuracy in regression analyses, these data suggest that left-handers’ superior performance stems from an associated right cerebral hemisphere advantage for visuospatial memory processing. As left handers showed superior visuospatial memory performance, and stronger visuospatial function has been linked to better motor skill retention in older adults (Lingo VanGilder et al., 2018; 2021), the present results have potential implications for rehabilitative efficacy.

RCFT copying performance was not associated with handedness in this older adult sample, with left-, mixed- and right-handed participants all producing highly accurate copies of the complex figure. This finding is important as it establishes that handedness was not associated with these participants’ visuo-constructive or drawing skills. The equivalent performance for RCFT copying thus provides a meaningful baseline against which recall performance can be compared; any differences observed in recall must, of necessity, reflect differences in visual memory performance in the absence of differences in visual motor copying.

The results revealed that left-handed older adults demonstrated superior RCFT immediate and delayed visual spatial recall. Handedness showed a highly significant relationship to both immediate and delayed recall performance, with left-handers gaining higher scores than either mixed- or right-handers. Thus, there was the same left-handed advantage for visuospatial memory in this sample as has been shown previously in younger adults (Rakhmavov & Dane, 2020). The visuospatial memory advantage likely reflects greater right hemisphere involvement in left- than right-handers’ processing. Because the left hand is predominantly controlled by the right cerebral hemisphere (Patten, 1996), and the right hemisphere is dominant for visuospatial processing (Corballis, 2003), left-handers demonstrate performance advantages across a broad range of visuospatial tasks (Annett, 2002). Previous research has established that this visuospatial processing superiority extends to right-handers who have a family history of left-handedness (e.g., Casey et al., 1990; D’Andrea & Spiers, 2005; Weinstein et al., 1990), consistent with the notion that the genes that predispose left-handedness are linked to a pattern of brain organization that favors visuospatial processing. Theoretically then, the visual memory advantage previously reported for young adults (Rakhmavov & Dane, 2020) should extend into older adulthood, as confirmed in the present investigation.

Importantly, as both age and intelligence were entered as covariates in the models, the left-handed visual memory advantages observed cannot be attributed to these potential confounds. Given that visuospatial memory has previously shown a significant deterioration with age, declining at twice the rate of verbal memory (Murre et al., 2013), these findings instead suggest that left handedness may confer a protective advantage for visual memory in older adults that can be detected by RCFT recall performance (Lingo VanGilder et al., 2021).

Research has yet to examine whether handedness is a reliable predictor of rehabilitation outcomes. However, the present findings imply that left-handers’ superior visual memories may auger greater success: recent research has found that RCFT delayed recall performance predicts motor skill retention in older adults (e.g., Lingo VanGilder et al., 2018; 2021). During motor rehabilitation the repetitive practice of functional movement patterns is used to induce learning (or relearning) of novel motor skills (Anderson et al., 2021). LingoVanGilder et al. (2022) demonstrated that both retention of these motor skills and stronger visuospatial memory are associated with greater white matter integrity in regions linking the frontal and parietal cortices (e.g., corticospinal tract, superior longitudinal fasciculi). Consistently, previous research has reported differences in the structure of these white matter tracts in left- and right-handers (e.g., Budisavljevic et al., 2021; Seizur et al., 2014). Given the roles of these tracts in supporting hand movement (the corticospinal tract is important for motor execution and manual dexterity; the superior longitudinal fasciculi are involved in visuomotor control during hand movements; Budisavljevic et al., 2021), handedness differences make intuitive sense. Left-handers’ greater reliance on the right hemisphere thus appears to confer a visuospatial memory advantage, reflecting the right hemisphere’s dominance for visuospatial attention and processing (e.g., Mengotti et al., 2020). In sum, as RCFT delayed recall performance predicts upper-extremity (i.e., hand, wrist and arm) skill learning in older adults (Lingo VanGilder et al., 2021), and the present study indicates superior RCFT delayed recall in left-handed older adults, it appears probable that left-handers would exhibit greater responsiveness to motor rehabilitation and higher integrity of the white matter tracts supporting visuospatial memory and motor skill retention. However, more research is needed to confirm this speculation.

It is worthy of note that both covariates were highly significant predictors of both the copying and recall components of RCFT performance. As anticipated, results confirmed that the older adults’ performance was influenced by their age, with participants at the older end of the older adult range performing less accurately than those at the younger end of the scale. In contrast, intelligence was a positive predictor of RCFT performance, with increasing TOPF IQ scores associated with greater accuracy for copying, immediate recall, and delayed recall. These findings appear entirely consistent with those of past researchers in which visual memory increased with increasing IQ scores (Gallagher & Burke, 2007) and declined with increasing age (Murre et al., 2013). As such, the present results reaffirm the need to control for these potential confounds when investigating the correlates of visual memory.

### Limitations and Directions for Further Research

The present sample included 62 left handers (7.77%), 27 mixed handers (3.39%) and 708 right handers (88.83%). This distribution of handedness scores appears broadly consistent with that seen in Briggs and Nebes’ (1975) original study (9.13% left handers, 5.25% mixed handers, 85.62% right handers), and the general population, suggesting that the sample is representative in terms of handedness. Approximately 10% of the general population is left-handed, with individual studies reporting 4%–18% left-handers; differences in proportions have been attributed to cultural and assessment differences (Papadatou-Pastou et al., 2020; Willems et al., 2014). However, it is important to acknowledge that the gender split of the current sample was skewed, with 152 females (19.07%) and 645 males (80.93%). As the Whitehall II study was specifically designed to recruit employees of the London civil service in the 1980s, this gender bias is not surprising. Since the 1980’s much work has been done to ensure that the UK civil service better reflects the diversity of the population, and the proportion of female employees had now risen to 54.02% (Civil Service Diversity and Inclusion Dashboard, 2022). Fortunately, the Whitehall II’s large sample size mitigates the gender skew, with the analyses confirming that gender did not influence RCFT recall or copying performance and did not interact with handedness, consistent with previous research in younger adults (Rakhmavov & Dane, 2020).

TOPF IQ was entered as a covariate in all the regression models to ensure that any observed visual memory effects could not be attributed to differences in intelligence (e.g., Gallagher & Burke, 2007). That said, it is important to note that the present sample’s mean TOPF IQ (*M* = 118.5, *SD* = 10.08) was higher than average, impacting the generalizability of the findings. Though the range of TOPF IQ scores represented in the sample was broad (75 – 151), the fact that the mean score was close to two SDs higher than average indicates that the sample is not representative of the wider population in terms of intelligence. As such, further research is needed to determine whether the visual memory advantage observed for left-handed older adults in the present sample is similarly observed in older adults who have lower IQs.

While the Whitehall II study is longitudinal, the present analyses were necessarily cross-sectional: handedness data were only collected as part of the Whitehall II MRI sub-study. Whilst cross-sectional studies have been a dominant research paradigm in aging research (Hofer et al., 2002), they present several limitations that may impact generalizability. Cross-sectional designs are vulnerable to cohort effects, with participants having experienced different historical and cultural influences that potentially affect their behavior and development (Schaie, 2013). For example, participants in the Whitehall II study were all aged 35–55 in 1985–1988 and so grew up in a time where being left-handed was still being actively discouraged (e.g., Peto, 1994). As such, the proportion of left handers and the strength of hand preference in this sample may be lower than would be anticipated from a cohort born later, when it was (and is) far less common to prohibit left handedness in schools. Thus, while the present study was the first to examine the associations between handedness and visual memory in older adults, further research is needed to a) confirm the present findings in other older adult cohorts, and b) examine the trajectory of the association between handedness and visual memory longitudinally.

## Conclusion

Whilst left handedness has been linked to a range of costs (see Fritsche & Lindell, 2019), these data highlight a visuospatial memory advantage for left-handers in older age. The present findings confirm that the left-handed superiority for immediate and delayed visual spatial recall that has been evident in young adults (Rakhmavov & Dane, 2020) persists into older adulthood, with left-handed older adults demonstrating visual memory advantages when compared with either mixed- or right-handers. As RCFT delayed recall performance is a strong predictor of rehabilitation efficacy in older adults (Lingo VanGilder et al., 2021), these findings suggest that left-handers’ stronger retention of visual spatial skills may auger greater success in motor rehabilitation. As previous research assessing the links between visuospatial memory and rehabilitation efficacy has examined only right-handed older adults, further research is needed to confirm this speculation.
